# Consumer-Reported Adverse Health Events and Product Problems Associated with E-Cigarettes: Evidence from FDA Tobacco Product Problem Reports

**DOI:** 10.1177/1179173X261491557

**Published:** 2026-09-18

**Authors:** Atia Daqiq, Steven Barrientos, Do Hong Nguyen, Ebbie Kalan, Linnea Laestadius, Shikha Prashad, Ziyad Ben Taleb

**Affiliations:** 1 Department of Kinesiology, College of Nursing and Health Innovation, 12329 The University of Texas at Arlington, Arlington, TX, USA; 2 Department of Behavioral and Community Health, School of Public Health, 1068 University of Maryland, College Park, MD, USA; 3 Zilber College of Public Health, University of Wisconsin–Milwaukee, Milwaukee, WI, USA

**Keywords:** E-cigarettes, ENDS, Adverse Health Events, Product Issues, FDA, Safety Reporting Portal, Tobacco Product Problem Reports, Post-market Surveillance, Consumer Reports, Tobacco Regulatory Science

## Abstract

**Background:**

The U.S. Food and Drug Administration (FDA) monitors potential health risks associated with electronic cigarettes (e-cigarettes) through Tobacco Product Problem Reports (TPPRs), which document adverse health events (AHEs) and product-related problems. This study characterized AHEs and product problems associated with e-cigarette use reported in TPPRs from 2017 to 2025.

**Methods:**

We analyzed 883 TPPRs. Descriptive statistics summarized report- and AHE-level frequencies and percentages across predefined health categories, while exploratory analyses assessed temporal trends in AHE and product-problem distributions. Categories included respiratory, neurological, psychological, cardiovascular, oral/dental, integumentary, musculoskeletal, digestive, addiction, physical trauma, death, laboratory tests or treatments, and nonspecific symptoms. Product problems were classified using FDA Safety Reporting Portal categories to identify commonly reported issues.

**Results:**

Reports most frequently contained neurological AHEs (n=418, 47.3%), followed by respiratory (n=336, 38.1%), nonspecific (n=196, 22.2%), digestive (n=93, 10.5%), and cardiovascular AHEs (n=86, 9.7%). Report-level AHE categories were not mutually exclusive. Less common categories included laboratory tests or treatments (n=63, 7.1%), physical trauma and psychological AHEs (n=59, 6.7% each), oral/dental (n=50, 5.7%), addiction (n=32, 3.6%), musculoskeletal (n=22, 2.5%), integumentary (n=14, 1.6%), and death (n=13, 1.5%; reported by secondary individuals). Device malfunctions were the most frequently reported product problem category (n=131, 26.3%), followed by packaging issues (n=118, 23.6%), other or unspecified issues (n=102, 20.4%), and sensory-related product issues (n=87, 17.4%). Less frequently reported problems included labeling and marketing concerns (n=34, 6.8%), contamination or foreign material (n=17, 3.4%), and counterfeit or unauthorized products (n=10, 2.0%). Common examples included device malfunctions resulting in explosions, packaging damage, taste complaints, labeling errors, incorrect products, and foreign material contamination. Temporal analyses identified increasing psychological AHEs, decreasing device malfunctions, and increasing packaging issues over time.

**Conclusion:**

This analysis highlights frequently reported neurological and respiratory AHEs and serious product safety concerns, underscoring the need for continued surveillance and regulatory oversight of e-cigarettes.

## Introduction

In the United States (U.S.), the 2009 Family Smoking Prevention and Tobacco Control Act granted the U.S. Food and Drug Administration (FDA) authority to regulate the manufacture, marketing, sale, and distribution of tobacco products.^1^ Through its Center for Tobacco Products, the FDA receives reports of tobacco product-related problems and adverse health events (AHEs) through the Safety Reporting Portal (SRP). These reports are primarily provided by consumers, but may also be submitted by health professionals, manufacturers, and other stakeholders.^2^ These reports are compiled into Tobacco Product Problem Reports (TPPRs) and released publicly in quarterly summaries available via the FDA website and the openFDA database.^3^ TPPRs are an important source of real-world surveillance data that may support the early detection of potential product problems and recurring safety concerns.

Electronic cigarettes (e-cigarettes), also referred to as electronic nicotine delivery systems (ENDS), are among the most commonly used tobacco products in the U.S. In 2024, 7% of U.S. adults reported current e-cigarette use (i.e., on some days or every day), with the highest prevalence among adults aged 18–24 years (14.8%).^4^ Use among young adults is particularly concerning, as 71.5% of current e-cigarette users aged 18–20 years had never used combustible cigarettes.^5^ This pattern raises concerns about nicotine dependence, dual use with cigarettes (reported by about 29% of adults who use e-cigarettes), and prolonged nicotine exposure during early adulthood.^6^ At the same time, the e-cigarette marketplace has rapidly evolved with newer disposable devices capable of delivering nicotine more efficiently and at higher concentrations.^7^ Despite their widespread use and evolving designs, e-cigarettes are often perceived as less harmful than combustible cigarettes. In one study, 57.3% of e-cigarette users reported lower perceived harm as a primary reason for their use.^8^ Together, the widespread use, evolving device designs, and lower perceived harm of e-cigarettes underscore the need for ongoing surveillance of AHEs and product-related problems.

Recent systematic reviews have reported associations between e-cigarette use and a range of AHEs affecting multiple body systems, including respiratory, neurological, and cardiovascular.^9-11^ However, AHEs reported through consumer-submitted regulatory surveillance systems remain less well characterized, particularly for e-cigarettes. Most prior studies have relied on data collected in research or clinical settings, with less attention to consumer-submitted reports captured through post-market surveillance systems. Although prior work has described AHEs reported through TPPRs across tobacco and nicotine products more broadly, examinations of e-cigarette-specific AHEs within TPPR data remain limited.^12^

Given the continued popularity of e-cigarette use, especially among young adults, and the limited characterization of reported e-cigarette AHEs, analysis of regulatory surveillance data is needed. In this study, we analyzed TPPRs submitted to the FDA between 2017 and 2025 to describe reported AHEs and product-related problems associated with e-cigarettes. Specifically, we aimed to (1) describe the distribution and frequency of reported AHEs; (2) assess temporal trends in reporting; and (3) identify recurring device- or product-related problems. By using publicly available consumer-reported data, this study contributes to our understanding of real-world e-cigarette–associated harms and may help inform regulatory oversight, public health surveillance, and future research.

## Methods

### Study Design and Data Source

This retrospective descriptive study analyzed e-cigarette-related reports from the U.S. FDA TPPRs. TPPR datasets covering January 1, 2017, through August 31, 2025, were obtained from the FDA website and downloaded between September 8, 2025, and September 30, 2025.^3^ Quarterly TPPR datasets were merged into a single master dataset using unique report identification numbers. Variables used from the TPPR datasets included report identification number, submission date, number and type of tobacco products reported, number and description of reported health problems, whether a nonuser was affected, and number and description of reported product problems. The initial merged dataset contained 1,430 reports. As this study utilized publicly available, de-identified data, Institutional Review Board review was not required.

### Eligibility Criteria and Data Cleaning

Reports were eligible for inclusion if (1) the reported product was identified as an e-cigarette or ENDS, (2) the report described at least one AHE, and (3) sufficient narrative detail was available to permit AHE classification. Reports were excluded if they reported no AHEs, involved non-e-cigarette tobacco products (e.g., combustible cigarettes, cigars, smokeless tobacco), reflected mixed-product use involving an e-cigarette and another tobacco or nicotine product when the specific contribution of e-cigarette exposure could not be determined, or contained missing, incomplete, or insufficient information needed to determine eligibility or classify the AHE.

Of the 1,430 reports in the initial dataset, 271 were excluded because they did not describe any AHEs. The remaining 1,159 reports underwent further review, and 276 additional reports involving non-e-cigarette tobacco products, mixed-product use, or insufficient information for final classification were excluded. The final sample included 883 exclusive e-cigarette reports. A data-cleaning flow diagram showing the inclusion and exclusion process is presented in Figure 1. Narrative text fields were evaluated to confirm e-cigarette exposure and verify that at least one codable AHE was present. When multiple AHEs were described within a narrative, each discrete AHE was separated, coded independently, and included in the AHE-level analysis without prioritization.

**Figure 1.**
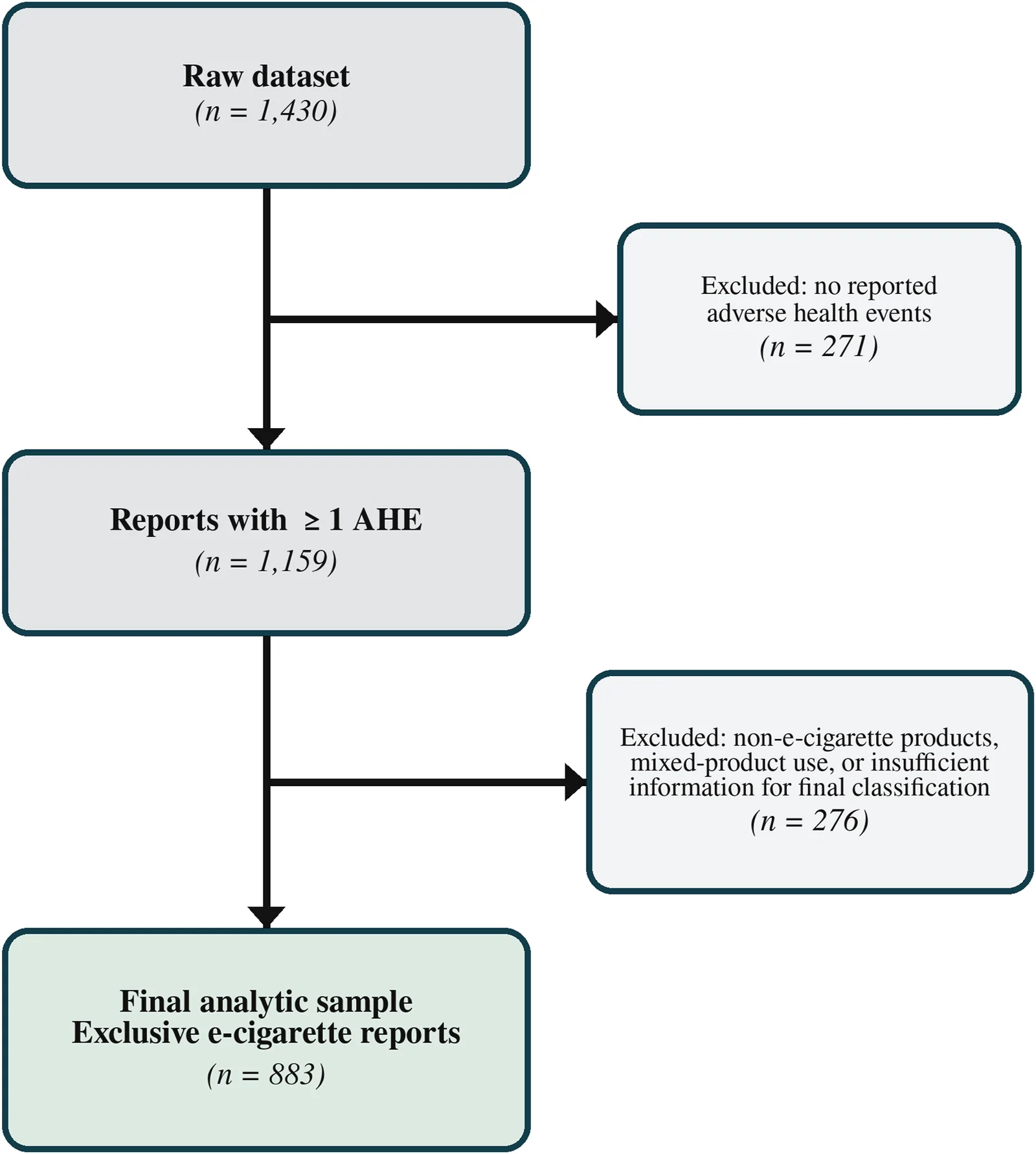
Data-cleaning flow diagram for included e-cigarette TPPRs

### Adverse Health Event Coding and Classification

AHEs were identified primarily from free-text narrative descriptions and classified using a coding framework based on International Classification of Diseases (ICD-10) codes.^13^ Each discrete AHE was reviewed and assigned to one of the following health categories: respiratory, psychological, neurological, cardiovascular, oral/dental, digestive, musculoskeletal, integumentary, laboratory tests or treatments, addiction, physical trauma, death, or nonspecific. The “laboratory tests or treatments” category captured AHEs involving testing, laboratory findings, procedures, or therapies. Categories were defined to group clinically similar AHEs into broader body system–based classifications, with working definitions provided in Appendix 1. For example, AHEs such as “breathing abnormally deep”, “cough”, and “chronic obstructive pulmonary disease” were categorized as “respiratory”. AHEs including “emotional distress”, “fear”, and “anxiety” were categorized as “psychological”. Categories were mutually exclusive at the AHE level, meaning each discrete AHE was assigned to only one health category. However, individual reports could contain multiple AHEs spanning different health categories. Events that could not be clearly assigned to a specific health category were coded as “nonspecific”, as defined in Appendix 2. After coding was completed, 2,135 AHEs were identified across the 883 included reports.

### Interrater Reliability for AHE Classification

To assess the reliability of AHE classification and reduce potential bias, a random 10% subset of reports (n = 90) was independently coded by a second researcher using the established coding framework. This process was used to evaluate agreement between coders and strengthen the consistency of the coding procedure.^14,15^ Discrepancies were reviewed and resolved through discussion and consensus; a third researcher was consulted when needed. Interrater reliability for AHE classification across all AHE categories was assessed on the double-coded subset using Cohen’s weighted kappa.^15^ The average interrater reliability across AHE categories was 0.979, with values ranging from 0.883 to 1.000, indicating an almost perfect level of agreement between the two raters. Detailed interrater reliability results are presented in Appendix 3.

### Product-Related Problem Classification

Product-related problems were coded separately from AHEs using FDA SRP “Product Problem Type” classifications.^16^ Coding was based on both the structured product problem selections available in the SRP and any accompanying free-text descriptions provided in the report. Unlike AHEs, which captured reported health outcomes, product-related problems captured issues with the product itself. These detailed product problem types were grouped into broader analytic categories: device malfunction, product packaging issues, labeling or marketing concerns, sensory-related product issues, contamination or foreign material (i.e., material in the product that did not belong), counterfeit or unauthorized product, and other/unspecified product issues. Multiple product-related problems could be coded within a single report when more than one problem was described. In total, 499 product-related problems were identified across the included reports.

### Statistical Analysis

Descriptive analyses were conducted at both the report level (n = 883) and the AHE level (n = 2,135). Frequencies and percentages were calculated to summarize AHEs across health categories and product-related problem types. For report-level AHE analyses, percentages were based on the 883 included reports and were not mutually exclusive. For AHE-level and product-related problem analyses, percentages were based on the total number of coded AHEs or product-related problems, respectively. Temporal patterns were assessed using yearly counts and within-year percentages. Because 2025 data were incomplete, inferential trend analyses were restricted to complete calendar years 2017–2024, with 2025 included in sensitivity analyses. Overall differences in AHE and product-problem distributions by year were assessed using Pearson chi-square tests with Monte Carlo p values (100,000 replicates) because of sparse expected cell counts; Cramer’s V was used to estimate effect size. Category-specific linear trends were examined using separate binomial logistic regression models, with calendar year modeled continuously and each category compared with all other coded events. Results are reported as odds ratios (ORs) per one-year increase with 95% confidence intervals (CIs). The Benjamini-Hochberg procedure was applied separately to AHE and product-problem analyses to control the false discovery rate, with q < 0.05 considered statistically significant. These exploratory analyses characterize reporting patterns and do not estimate population incidence or risk. Data management and statistical analyses were performed using Microsoft Excel, R 4.5.2, and IBM SPSS V.30.

## Results

### Distribution of AHE Categories

Among the 883 reports, neurological AHEs were most frequently reported (n = 418; 47.3%), followed by respiratory (n = 336; 38.1%), nonspecific (n = 196; 22.2%), digestive (n = 93; 10.5%), and cardiovascular AHEs (n = 86; 9.7%) (Figure 2). Less frequent categories included laboratory tests or treatments (n = 63; 7.1%), physical trauma and psychological (n = 59; 6.7% each), oral/dental (n = 50; 5.7%), addiction (n = 32; 3.6%), musculoskeletal (n = 22; 2.5%), integumentary (n = 14; 1.6%), and death (n = 13; 1.5%). Report-level percentages are not mutually exclusive.

**Figure 2.**
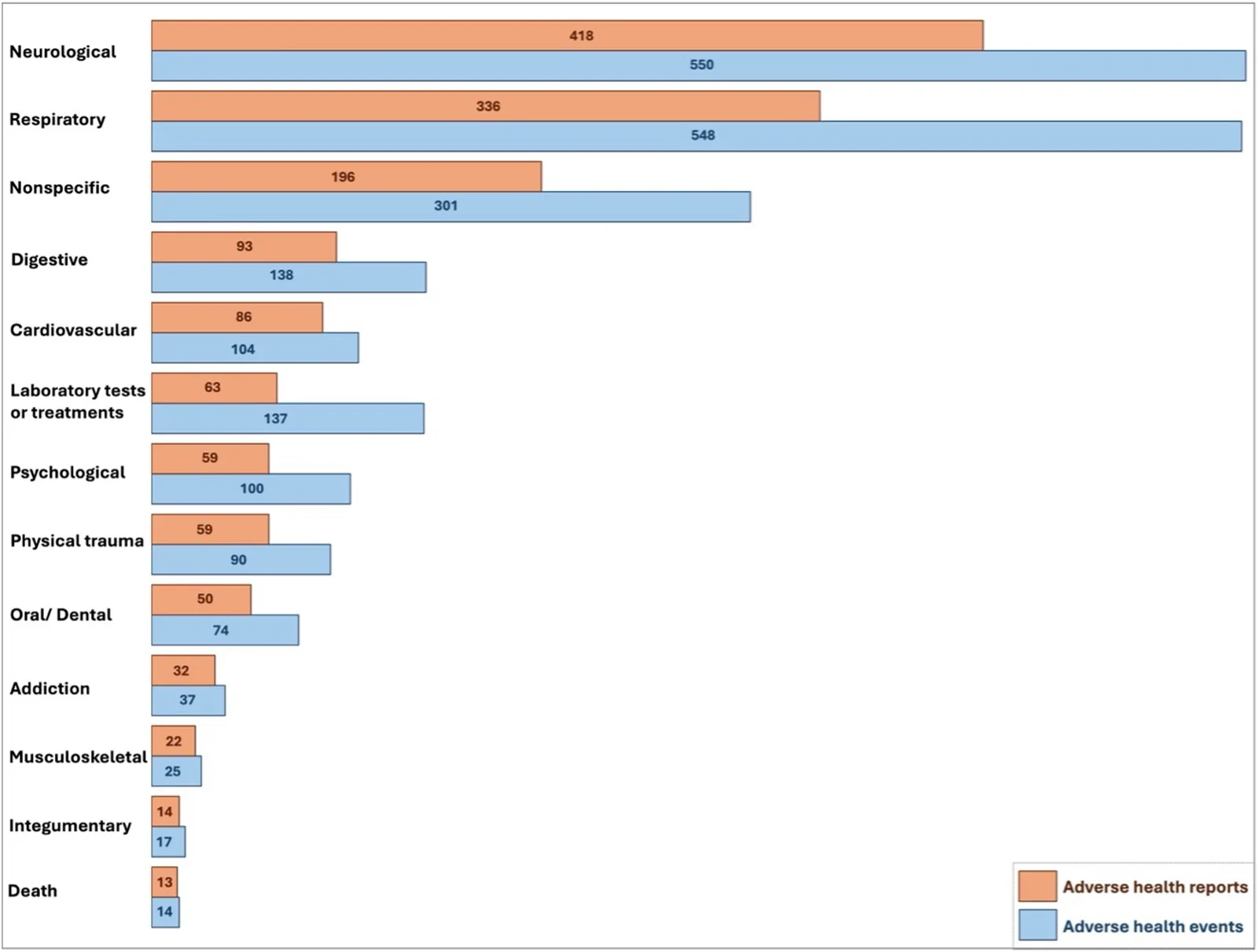
E-cigarette-related adverse health reports and adverse health events by health category, 2017–2025

Across all discrete AHEs (n = 2,135), neurological events were again the most frequent category (n = 550; 25.8%), closely followed by respiratory events (n = 548; 25.7%) (Figure 2). Nonspecific events were the third most common category (n = 301; 14.1%), followed by digestive (n = 138; 6.5%) and laboratory tests or treatments (n = 137; 6.4%). Other AHEs included cardiovascular (n = 104; 4.9%), psychological (n = 100; 4.7%), physical trauma (n = 90; 4.2%), oral/dental (n = 74; 3.5%), addiction (n = 37; 1.7%), musculoskeletal (n = 25; 1.2%), integumentary (n = 17; 0.8%), and death (n = 14; 0.7%).

### Product-Related Problem Categories

Figure 3 presents product-related problems grouped into broader analytic categories based on FDA SRP product problem classifications. A total of 499 product-related problems were identified. Device malfunction was the most frequently reported product problem category (n = 131; 26.3%), followed by product packaging issues (n = 118; 23.6%), other or unspecified product issues (n = 102; 20.4%), and sensory-related product issues (n = 87; 17.4%). Less frequent categories included labeling or marketing concerns (n = 34; 6.8%), contamination or foreign material (n = 17; 3.4%), and counterfeit or unauthorized product concerns (n = 10; 2.0%). Within the device malfunction category, common problem types included explosions (n = 38; 7.6%), fires (n = 29; 5.8%), overheating (n = 23; 4.6%), product failure (n = 21; 4.2%), and abnormal burning after being lit (n = 20; 4.0%). Packaging-related issues included child-safety hazards (n = 29; 5.8%), leaking (n = 28; 5.6%), damaged or defective products (n = 24; 4.8%), and spilling (n = 14; 2.8%). Sensory-related product issues most often involved taste (n = 40; 8.0%), appearance or look (n = 27; 5.4%), and smell (n = 20; 4.0%).

**Figure 3.**
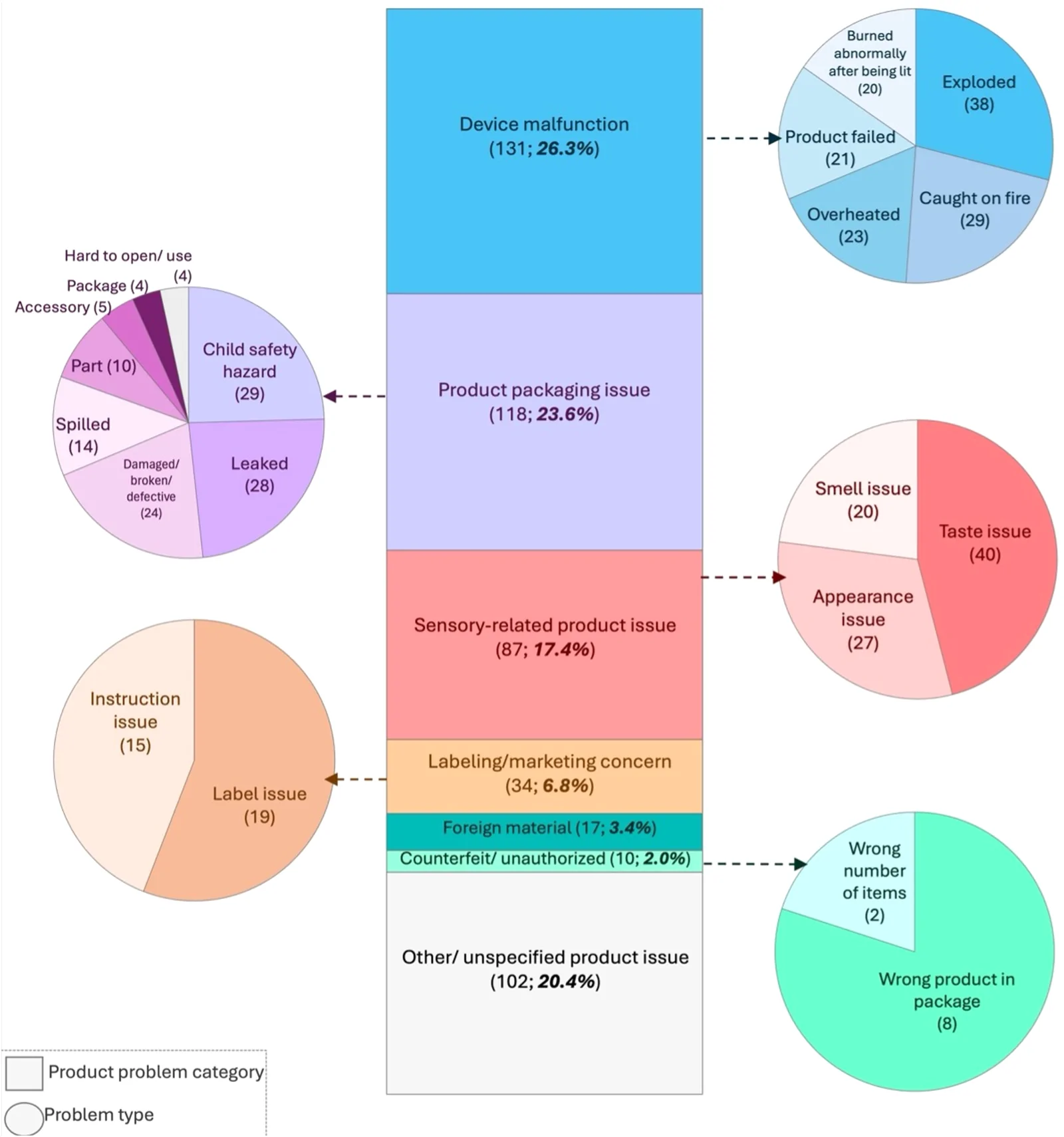
Characteristics of e-cigarette-related product problems, 2017–2025

### Temporal Trends in Reports, AHE Categories, and Product Problems

Reports were concentrated in 2019 (n = 538; 60.9%), with the highest number occurring in the third quarter (Q3) of 2019 (n = 294; 33.3%). Reporting declined after 2019 and remained lower throughout the later years of the study period.

The annual distribution of AHE categories is shown in Appendix 4. Neurological and respiratory AHEs represented the largest proportions of coded AHEs across several years, although the distribution varied over time. Respiratory AHEs accounted for 28.8% of coded AHEs in 2019 and remained prominent in 2023 (25.2%) and 2025 (29.2%). Neurological AHEs increased from 9.3% in 2017 to a peak of 35% in 2021, remained high in 2022 (31.9%), and declined to 12.9% in 2024. Other categories showed more year-specific patterns: nonspecific AHEs reached their highest proportion in 2024 (35.3%), physical trauma accounted for larger shares in 2017 and 2018 (25.9% and 23.4%, respectively), and psychological AHEs were highest in 2023 (15.4%). Digestive, cardiovascular, laboratory tests or treatments, oral/dental, addiction, musculoskeletal, integumentary, and death-related events generally represented smaller proportions of coded AHEs across the study period.

The annual distribution of product-related problem categories is shown in Appendix 5. Product-related problems varied across the study period, with the highest number reported in 2019 (n = 154; 30.9%), followed by 2018 (n = 77; 15.4%) and 2017 (n = 69; 13.8%). Using within-year percentages, device malfunctions accounted for the largest share of reported product problems in 2017 and 2018 (46.4% and 45.4%, respectively) but declined in later years. In contrast, product packaging issues became more prominent over time, reaching 32.6% in 2022 and 44.4% in 2024. Other or unspecified product issues fluctuated across years, peaking at 36.4% in 2019 and remaining among the more frequently reported categories in several later years. Sensory-related product issues generally accounted for 11.6% to 23.3% of reported problems but increased to 44.4% in 2025. Labeling or marketing concerns, contamination or foreign material issues, and counterfeit or unauthorized product concerns consistently represented smaller proportions of reported product problems across the study period.

Across complete calendar years 2017-2024, the overall distribution of coded AHEs differed by year (Monte Carlo p < 0.00001; Cramer’s V = 0.155). After false discovery rate correction, the proportion of AHEs classified as psychological increased over time (OR per year = 1.26; 95% CI, 1.13-1.40; q < 0.001). Product-problem distributions also differed by year (Monte Carlo p < 0.00001; Cramer’s V = 0.191). The proportion of product problems classified as device malfunctions decreased (OR per year = 0.86; 95% CI, 0.78-0.95; q = 0.016), whereas the proportion classified as product packaging issues increased (OR per year = 1.15; 95% CI, 1.05-1.27; q = 0.016). All three trends remained statistically significant in sensitivity analyses that included partial-year 2025 data.

## Discussion

This study characterized AHEs and product-related problems reported in FDA TPPRs involving exclusive e-cigarette use from 2017 through 2025. Across 883 reports, 2,135 discrete AHEs were identified. Neurological and respiratory events were the most frequently reported AHE categories, together accounting for more than half of all coded AHEs. Device malfunction and packaging-related concerns were the leading product problems. These findings build on prior TPPR research by focusing specifically on exclusive e-cigarette reports and by examining both reported AHEs and product problems.^2,12^

Neurological events were the leading AHE category, consistent with prior TPPR research in which neurological symptoms were also among the most frequently reported AHE categories.^12^ This pattern is notable because e-cigarettes can deliver nicotine rapidly, and newer high-nicotine or salt-based products may increase nicotine exposure compared with earlier device generations.^7^ Nicotine acts on nicotinic acetylcholine receptors in the central nervous system and, at high levels, may increase neuronal excitability and contribute to neurological symptoms such as dizziness, tremors, syncope, or seizures.^17-19^ Exposure to nicotine may also weaken the integrity of the blood-brain barrier, potentially allowing greater penetration of toxins into the central nervous system.^20^ Although nicotine exposure has been shown to produce short-term cognitive benefits in nonsmokers, such as improvements in attention and working memory,^21^ these transient effects stand in contrast to evidence of longer-term neurological consequences. For example, greater cumulative nicotine exposure has been associated with reduced brain volumes, suggesting a dose-related relationship with neurodegeneration.^22^ Neuroimaging studies further indicate that nicotine dependence is linked to disruptions in white matter functional networks, including reduced efficiency of localized (i.e., segregated) processing, while largely preserving broader network integration.^23^ In addition, nicotine exposure has been independently associated with white matter hyperintensities, with periventricular regions more affected in young adults and deeper white matter regions more affected in older populations.^24^ These structural changes, such as white matter hyperintensities and subcortical atrophy, have been associated with poorer executive function and greater global cognitive decline.^25,26^ E-cigarette aerosols may contain neurotoxic metals released from device components, including lead, chromium, nickel, and manganese, which have been linked to oxidative stress, mitochondrial dysfunction, and neuroinflammatory pathways.^27,28^ Damage related to oxidative stress is also strongly associated with glutamate-driven excitotoxic mechanisms, which can further exacerbate neural injury. These mechanisms provide biologically plausible pathways that may help explain the neurological symptoms reported in e-cigarette surveillance data and support the need for continued monitoring of neurological outcomes. This need is especially urgent given that a separate national study showed that adults with epilepsy have significantly higher rates of exclusive e-cigarette and dual tobacco use (e-cigarettes and cigarettes) compared with those without the condition.^29^ Furthermore, current e-cigarette use was independently associated with higher odds of having epilepsy, highlighting compounding risks for a demographic already highly vulnerable to seizures.^29^

Respiratory events were nearly as frequent as neurological events, which is consistent with the broader e-cigarette literature. Prior studies have linked e-cigarette use with cough, wheezing, shortness of breath, chest discomfort, and airway irritation.^30,31^ These symptoms may reflect exposure to aerosol constituents such as propylene glycol, vegetable glycerin, flavoring chemicals, carbonyl compounds, and metals, which can irritate the airways and contribute to oxidative stress and pulmonary inflammation.^30-32^ The wide variability in flavoring formulations may also contribute to differences in toxicological profiles across e-cigarette products.^33^ These findings are relevant in the context of e-cigarette or vaping product use-associated lung injury (EVALI), an outbreak of serious acute lung injury associated with vaping-related exposures, particularly tetrahydrocannabinol (THC)-containing products with vitamin E acetate.^32,34^ Follow-up studies have also found that some patients with EVALI report persistent respiratory symptoms, reduced functioning, cognitive symptoms, or mood-related concerns after the acute illness period.^35^

The temporal pattern of reports provides important context. Reports were concentrated in 2019, with the highest number occurring in Q3 2019, followed by a decline in later years. This timing overlaps with the EVALI outbreak, when public and regulatory attention to vaping-related respiratory harms increased.^32,34^ A similar trend was observed in another TPPR study.^12^ The annual distribution of AHE categories showed that neurological and respiratory events represented large proportions of coded AHEs across several years, while other categories, including nonspecific, physical trauma, and psychological AHEs, showed more year-specific variation. Product-related problems also varied over time, with device malfunctions accounting for larger shares in earlier years and product packaging issues, other or unspecified product issues, and sensory-related product issues becoming more prominent in later years. Formal trend analyses showed an increasing proportion of psychological AHEs over time, while the proportion of product problems classified as device malfunctions decreased and the proportion classified as product packaging issues increased after false discovery rate correction. The e-cigarette market has evolved substantially over the study period, including shifts toward disposable devices, larger e-liquid capacities, and higher nicotine concentrations.^36,37^ Importantly, nicotine concentration alone does not fully characterize nicotine exposure, which is also influenced by device characteristics and user behavior, including puffing patterns.^38^ These changes in product design and nicotine delivery may contribute to variation in the types of product-related problems reported. Overall, these patterns suggest that TPPR reporting may reflect both changes in the e-cigarette marketplace and periods of heightened public awareness.

Beyond neurological and respiratory events, TPPRs captured AHEs across several other categories, including nonspecific, digestive, cardiovascular, psychological, oral/dental, physical trauma, and addiction-related events, among others. This pattern suggests that consumer-reported e-cigarette concerns may extend across multiple body systems, not only the respiratory tract. Some of these reports may reflect systemic exposure to nicotine and other aerosol constituents, while others may represent less well-characterized symptoms that are difficult for consumers to describe or classify.^11,27,28^ The high frequency of ‘nonspecific’ AHEs is therefore important, as it highlights both the breadth of reported AHEs and the challenges of interpreting consumer-reported surveillance data. At the same time, reports on digestive, cardiovascular, and psychological outcomes point to areas that remain underexplored and warrant further investigation.

Product-related problems add another dimension to the safety concerns found in this study. Device malfunction was the most common product problem category, including overheating, explosions, fires, abnormal burning, and product failure. Unlike AHEs related to aerosol exposure, these events represent acute injury through burns, blast trauma, or contact with malfunctioning device components. Prior studies have reported e-cigarette explosions and burn injuries treated in emergency departments, and case reports describe severe burns, facial trauma, dental injury, fractures, and injuries requiring surgical treatment.^39,40^ These events are often linked to lithium-ion battery failures, improper charging, unsafe storage, or device defects.^41^ Therefore, TPPR reports involving explosions, fires, or overheating should be interpreted not only as product quality concerns, but also as potential contributors to acute physical injury.

Packaging and design problems further show that e-cigarette-related risks are not limited to aerosol exposure. Reports of leaks, spills, defective parts, and child-safety hazards point to issues with product contamination, product usability, and unintended exposure to nicotine or other harmful agents. Leaking/spilled e-liquids may increase the chances of nicotine exposure through skin contact or accidental ingestion, especially among children or nonusers. Reports involving abnormal taste, smell, appearance, contamination, foreign material, or counterfeit products may also indicate manufacturing inconsistencies, product degradation, or potential gaps in product quality oversight. These findings suggest that post-market surveillance should consider both reported health outcomes and product-related concerns. For e-cigarettes, the product itself, not only the aerosol it produces, may contribute to harm.

These findings have implications for e-cigarette surveillance and regulatory oversight. The frequent reporting of neurological and respiratory AHEs, together with device malfunctions and packaging-related problems, highlights the value of monitoring both health outcomes and product-specific concerns. Given the continued evolution of e-cigarette devices and formulations, post-market surveillance should be an ongoing process that draws on complementary sources, including adverse-event reporting systems, poison control data, national surveillance programs, and other real-world evidence, to identify emerging safety signals.^2,42,43^ More complete reporting of product characteristics, contents, co-use of other substances, and clinical outcomes could further improve the ability of surveillance systems to characterize these signals and inform regulatory responses as e-cigarette products continue to evolve. Such surveillance findings may also help prioritize areas for regulatory evaluation, including product authorization, product standards, labeling and risk communication, and post-market monitoring.^1^ Because device designs and formulations change rapidly, regulatory evaluation should remain responsive to emerging technologies rather than assuming that evidence from earlier products applies directly to newer generations.^36,37^

Although heated tobacco products (HTPs) are distinct from e-cigarettes, evidence from other emerging nicotine products provides relevant regulatory context. In Spain, HTP adoption followed diffusion patterns similar to those observed for cigarette products perceived as healthier alternatives, suggesting that reduced-harm perceptions may influence product uptake.^44^ Market-level analyses also found that IQOS/Heets sales largely substituted for sales of conventional cigarettes marketed by the same manufacturer.^45^ Together, these findings further highlight the importance of product-specific surveillance and cautious communication of reduced-harm claims as novel nicotine products continue to evolve.

Future research should build on these findings by improving characterization of e-cigarette exposure, including device type, liquid formulation, nicotine delivery, frequency and duration of use, and use of other tobacco or nicotine products. Studies should also distinguish exclusive e-cigarette use from dual or polytobacco use, use standardized definitions of AHEs, and evaluate long-term effects using prospective study designs. Integrating surveillance data with clinical, toxicological, and epidemiological data may further strengthen interpretation of emerging safety concerns and help clarify potential causal relationships.

Several limitations should be considered. Because TPPRs are voluntary, consumer-submitted reports, potential issues include underreporting, selective or stimulated reporting, self-report and recall bias, and incomplete data, especially when unusual events (e.g., EVALI outbreak) receive public attention. Because the number of exposed e-cigarette users and the probability of making a report are unknown, these data cannot be used to estimate incidence or prevalence or determine causality. TPPRs do not provide systematic longitudinal follow-up, and medical confirmation and detailed clinical information were unavailable for many reports, limiting assessment of symptom progression and long-term outcomes. Accordingly, the limited long-term information captured in TPPRs should not be interpreted as evidence of long-term e-cigarette safety.

Product and exposure information was also incomplete and inconsistently reported, limiting our ability to account for differences in device characteristics, operating conditions, nicotine concentration, flavoring agents, liquid composition, and patterns or duration of use. Although reports involving known mixed-product use in which the specific contribution of e-cigarette exposure could not be determined were excluded, incomplete reporting may have limited our ability to identify all co-use of other tobacco, nicotine, or cannabis products. In addition, AHEs were identified largely from narrative reports rather than standardized clinical assessments, which may have introduced variability in how symptoms and events were described and classified. Missing brand, product content, and geographic information further limited product-specific and geographic interpretation. Because TPPRs are collected through a U.S.-based FDA reporting system, the findings may not be directly generalizable to other countries with different products, regulatory environments, use patterns, and adverse-event reporting practices. Lastly, despite a formal coding structure and interrater reliability efforts, some subjective judgments were necessary when coding narrative reports, particularly nonspecific AHEs or those with limited detail. These limitations should be considered when interpreting the reported AHE and product problem patterns.

## Conclusion

This study characterized AHEs and product-related problems reported in FDA TPPRs involving exclusive e-cigarette use. Neurological and respiratory AHEs were the most frequently reported categories, although reported AHEs extended across multiple body systems. Device malfunctions, packaging issues, and leaks suggest that e-cigarette-related harms may extend beyond aerosol exposure to product design and quality issues. These findings highlight the importance of ongoing surveillance, particularly as new e-cigarette devices, formulations, and product designs emerge on the market. Improved reporting of product characteristics and contents, co-use of other substances, and clinical outcomes may strengthen surveillance and support regulatory efforts to protect public health.

## Supplemental Material

Supplemental Material - Consumer-Reported Adverse Health Events and Product Problems Associated With E-Cigarettes: Evidence From FDA Tobacco Product Problem Reports Supplemental Material for Consumer-Reported Adverse Health Events and Product Problems Associated With E-Cigarettes: Evidence From FDA Tobacco Product Problem Reports by Atia Daqiq, Steven Barrientos, Do Hong Nguyen, Ebbie Kalan, Linnea Laestadius, Shikha Prashad, Ziyad Ben Taleb in Tobacco Use Insights.
