# Supplementary material for: Consumer-Reported Adverse Health Events and Product Problems Associated with E-Cigarettes: Evidence from FDA Tobacco Product Problem Reports
Source: Tob Use Insights. 2026 Sep 18;19:1179173X261491557. doi: 10.1177/1179173X261491557 (PMC13724922; doi:10.1177/1179173X261491557)
Supplement: Supplementary file 1 [file sj-pdf-1-tui-10.1177_1179173X261491557.pdf]

SUPPLEMENTARY MATERIAL

Appendix 1. Health category definitions for categorizing adverse health events.
Definitions of each health category:

1. **Neurological**: Symptoms of, relating to, or affecting the nervous system, including sensory input, information processing, motor function, and nerve signaling. This category includes conditions involving the central nervous system (e.g., brain and spinal cord) and peripheral nervous system (e.g., autonomic and somatic nervous systems).
2. **Respiratory**: Symptoms of, relating to, or affecting respiration, breathing, gas exchange, and the organs and tissues of the respiratory system. This includes the upper respiratory tract (e.g., nose, nasal cavities, and sinuses) and lower respiratory tract (e.g., trachea, bronchi, bronchioles, alveoli, and lungs).
3. **Digestive:** Symptoms of, relating to, or affecting digestion, nutrient absorption, metabolism, or elimination of solid waste, and/or the organs and tissues of the digestive system. This includes the gastrointestinal tract (e.g., esophagus, stomach, intestines, rectum, and anus) and accessory digestive organs (e.g., liver, pancreas, and gallbladder).
4. **Cardiovascular:** Symptoms of, relating to, or affecting the circulation of blood and the organs and tissues of the cardiovascular system. This includes the heart, blood vessels (e.g., arteries, veins, and capillaries), and blood components (e.g., plasma and blood cells).
5. **Laboratory Tests or Treatments**: Involving diagnostic evaluation, clinical or laboratory testing, medical procedures, therapeutic interventions, or treatment administered by healthcare professionals or healthcare facilities.
6. **Psychological**: Symptoms of, relating to, or affecting mental, emotional, behavioral, or cognitive functioning. This includes disturbances involving mood, anxiety, perception, personality, trauma-related conditions, eating behaviors, substance use, and other psychiatric or psychological disorders.
7. **Physical Trauma:** Symptoms or injuries resulting from external physical force, impact, violence, accidents, or other traumatic events causing bodily harm, tissue damage, or impairment.
8. **Oral/Dental**: Symptoms of, relating to, or affecting the mouth, teeth, gums, tongue, jaw, throat, or other oral and craniofacial structures involved in speaking, chewing, swallowing, or oral health.
9. **Addiction**: Symptoms of, relating to, or characterized by compulsive engagement in substances or behaviors despite harmful consequences, impaired control over use or activity, cravings, dependence, withdrawal symptoms, or functional impairment. This includes substance-related addictions (e.g., alcohol, nicotine, or drugs) and behavioral addictions (e.g., gambling).
10. **Musculoskeletal:** Symptoms of, relating to, or affecting body movement, support, stability, or posture, and/or the organs and tissues of the musculoskeletal system. This includes bones, muscles, joints, cartilage, ligaments, tendons, and connective tissues.
11. **Integumentary:** Symptoms of, relating to, or affecting the protective and regulatory functions of the skin and associated structures. This includes the skin

(e.g., epidermis, dermis, and subcutaneous tissue), hair, nails, sweat glands, and sebaceous glands.

12. **Death**: An individual who has sustained either (1) irreversible cessation of circulatory and respiratory functions, or (2) irreversible cessation of all functions of the entire brain, including the brain stem. This category does not include near-death experiences. Death events were coded when the death was described by a secondary reporter.
13. **Nonspecific:** Adverse health events, symptoms, or conditions that are generalized, nonspecific, multifactorial, or do not clearly fit within the defined categories above.

Appendix 2. Breakdown of the 'nonspecific' adverse health events category.
Adverse health events categorized as 'nonspecific' were further grouped by secondary descriptive categories:

1. **General health**: Abnormal weight gain, Anemia, Appetite lost, Bladder constriction, Cancer, Chills, Chemical poisoning, Cold, Cold symptoms, Cold feet, Cold hands & feet, Cold sweat, Excess sweating, Fatigue, Fatigue extreme, Feeling sick, Feels bad, Flu-like symptoms, Flu like symptoms, Flu, Feeling unwell, Fever, Generalized chest pain, Hot flashes, Infection parasitic, Illness, Ill feeling, Kaposi's sarcoma-lung, Low grade fever, Nonspecific chest pain, Pale skin, Starvation, Sweating, Idiopathic edema, Retinal vascular disorder NOS, Swelling, Urination involuntary, Pain.
2. **Autoimmune**: Allergy, Allergic shock.
3. **Eyes**, **nose, throat**: Bulging eyes, constricted pupils, Difficulty focusing eyes, Dry eye, Dryness of eyes, Eye ache, Nosebleed, Throat swelling, Nasal Pain, Vision abnormal.
4. **Environmental exposure**: Allergic reaction, Anaphylactic reaction, Anaphylactic shock, Device catching fire, Product explosion, Nicotine poisoning, Occupational environmental problem NOS.
5. **Legal/social Issues**: Apparent life threatening event, Criminal, Safety mechanism failed to activate.
6. **Metabolic/endocrine system**: Blood sugar increased, Diabetes, Hyperthyroidism, abnormal hormone levels, Loss of control of blood sugar, Metabolic disorder.
7. **Substance use**: E-cigarette smoker.
8. **Hearing/ears**: Earache.

Appendix 3. Cohen's weighted kappa for each health category.

Category	Cohen's Weighted Kappa [95% CI]	Std. Error	*K* Level of Agreement
Respiratory	1.000 [1.000, 1.000]	0.000	Perfect agreement
Psychological	0.917 [0.756, 1.000]	0.082	Almost perfect agreement
Neurological	1.000 [1.000, 1.000]	0.000	Perfect agreement
Cardiovascular	1.000 [1.000, 1.000]	0.000	Perfect agreement
Oral/Dental	0.883 [0.657, 1.000]	0.115	Almost perfect agreement
Integumentary	1.000 [1.000, 1.000]	0.000	Perfect agreement
Digestive	0.927 [0.786, 1.000]	0.072	Almost perfect agreement
Musculoskeletal	1.000 [1.000, 1.000]	0.000	Perfect agreement
Physical Trauma	1.000 [1.000, 1.000]	0.000	Perfect agreement
Death	1.000 [1.000, 1.000]	0.000	Perfect agreement
Lab Tests or Treatments	1.000 [1.000, 1.000]	0.000	Perfect agreement
Addiction	1.000 [1.000, 1.000]	0.000	Perfect agreement
Nonspecific	1.000 [1.000, 1.000]	0.000	Perfect agreement

Appendix 4. Annual distribution of adverse health event categories, 2017–2025.

**AHE Category (n=2,135)** / **Year**	**2017** n=54 ***(%)***	**2018** n=77 ***(%)***	**2019** n=1,359 ***(%)***	**2020** n=205 ***(%)***	**2021** n=117 ***(%)***	**2022** n=91 ***(%)***	**2023** n=123 ***(%)***	**2024** n=85 ***(%)***	**2025** n=24 ***(%)***
**Neurological**	5 ***(9.3)***	13 ***(16.9)***	354 ***(26.0)***	58 ***(28.3)***	41 ***(35.0)***	29 ***(31.9)***	32 ***(26.0)***	11 ***(12.9)***	7 ***(29.2)***
**Respiratory**	9 ***(16.7)***	7 ***(9.1)***	392 ***(28.8)***	56 ***(27.3)***	9 ***(7.7)***	21 ***(23.1)***	31 ***(25.2)***	16 ***(18.8)***	7 ***(29.2)***
**Nonspecific**	8 ***(14.8)***	11 ***(14.3)***	179 ***(13.2)***	25 ***(12.2)***	22 ***(18.8)***	9 ***(9.9)***	11 ***(8.9)***	30 ***(35.3)***	6 ***(25.0)***
**Digestive**	8 ***(14.8)***	5 ***(6.5)***	83 ***(6.1)***	17 ***(8.3)***	5 ***(4.3)***	6 ***(6.6)***	8 ***(6.5)***	5 ***(5.9)***	1 ***(4.2)***
**Cardiovascular**	1 ***(1.9)***	4 ***(5.2)***	72 ***(5.3)***	6 ***(2.9)***	8 ***(6.8)***	3 ***(3.3)***	8 ***(6.5)***	2 ***(2.4)***	0 ***(0.0)***
**Laboratory tests or treatments**	3 ***(5.6)***	3 ***(3.9)***	99 ***(7.3)***	19 ***(9.3)***	3 ***(2.6)***	3 ***(3.3)***	0 ***(0.0)***	7 ***(8.2)***	0 ***(0.0)***
**Physical trauma**	14 ***(25.9)***	18 ***(23.4)***	33 ***(2.4)***	5 ***(2.4)***	7 ***(6.0)***	4 ***(4.4)***	3 ***(2.4)***	6 ***(7.1)***	0 ***(0.0)***
**Psychological**	2 ***(3.7)***	6 ***(7.8)***	47 ***(3.5)***	7 ***(3.4)***	9 ***(7.7)***	3 ***(3.3)***	19 ***(15.4)***	6 ***(7.1)***	1 ***(4.2)***
**Oral/ dental**	1 ***(1.9)***	5 ***(6.5)***	49 ***(3.6)***	2 ***(1.0)***	4 ***(3.4)***	6 ***(6.6)***	6 ***(4.9)***	1 ***(1.2)***	0 ***(0.0)***
**Addiction**	0 ***(0.0)***	3 ***(3.9)***	21 ***(1.5)***	5 ***(2.4)***	3 ***(2.6)***	2 ***(2.2)***	2 ***(1.6)***	0 ***(0.0)***	1 ***(4.2)***
**Musculoskeletal**	0 ***(0.0)***	1 ***(1.3)***	17 ***(1.3)***	3 ***(1.5)***	1 ***(0.9)***	2 ***(2.2)***	1 ***(0.8)***	0 ***(0.0)***	0 ***(0.0)***
**Integumentary**	3 ***(5.6)***	1 ***(1.3)***	6 ***(0.4)***	1 ***(0.5)***	4 ***(3.4)***	1 ***(1.1)***	0 ***(0.0)***	0 ***(0.0)***	1 ***(4.2)***
**Death**	0 ***(0.0)***	0 ***(0.0)***	6 ***(0.4)***	2 ***(1.0)***	1 ***(0.9)***	2 ***(2.2)***	2 ***(1.6)***	1 ***(1.2)***	0 ***(0.0)***

Appendix 5. Annual distribution of product-related problem categories, 2017–2025.

Year / Product Problem Category (n=499)	2017 n=69 *(%)*	2018 n=77 *(%)*	2019 n=154 *(%)*	2020 n=27 *(%)*	2021 n=43 *(%)*	2022 n=46 *(%)*	2023 n=29 *(%)*	2024 n=36 *(%)*	2025 n=18 *(%)*
**Device malfunction**	32 ***(46.4)***	35 ***(45.4)***	21 ***(13.6)***	6 ***(22.2)***	10 ***(23.3)***	11 ***(23.9)***	8 ***(27.6)***	8 ***(22.2)***	0 ***(0.0)***
**Product packaging issue**	14 ***(20.3)***	10 ***(13.0)***	36 ***(23.4)***	7 ***(25.9)***	10 ***(23.3)***	15 ***(32.6)***	5 ***(17.2)***	16 ***(44.4)***	5 ***(27.8)***
**Sensory–related product issue**	8 ***(11.6)***	14 ***(18.2)***	25 ***(16.2)***	5 ***(18.5)***	10 ***(23.3)***	6 ***(13.1)***	5 ***(17.2)***	6 ***(16.7)***	8 ***(44.4)***
**Other/ unspecified product issue**	6 ***(8.7)***	5 ***(6.5)***	56 ***(36.4)***	8 ***(29.7)***	6 ***(13.9)***	7 ***(15.2)***	8 ***(27.6)***	4 ***(11.1)***	2 ***(11.1)***
**Labeling/ marketing concern**	5 ***(7.2)***	6 ***(7.8)***	13 ***(8.4)***	1 ***(3.7)***	3 ***(7.0)***	4 ***(8.7)***	0 ***(0.0)***	0 ***(0.0)***	2 ***(11.1)***
**Contamination/ foreign material**	2 ***(2.9)***	4 ***(5.2)***	0 ***(0.0)***	0 ***(0.0)***	3 ***(7.0)***	3 ***(6.5)***	3 ***(10.4)***	1 ***(2.8)***	1 ***(5.6)***
**Counterfeit/ unauthorized product**	2 ***(2.9)***	3 ***(3.9)***	3 ***(2.0)***	0 ***(0.0)***	1 ***(2.2)***	0 ***(0.0)***	0 ***(0.0)***	1 ***(2.8)***	0 ***(0.0)***
